# Celiac Disease and the Thyroid: Highlighting the Roles of Vitamin D and Iron

**DOI:** 10.3390/nu13061755

**Published:** 2021-05-21

**Authors:** Christina Starchl, Mario Scherkl, Karin Amrein

**Affiliations:** Department of Internal Medicine, Division of Endocrinology and Diabetology, Medical University of Graz, Auenbruggerplatz 15, 8036 Graz, Austria; c.starchl@medunigraz.at (C.S.); mario.scherkl@stud.medunigraz.at (M.S.)

**Keywords:** thyroid, celiac disease, Hashimoto’s thyroiditis, Grave’s disease, vitamin D, iron

## Abstract

Celiac disease (CD) and autoimmune thyroid diseases (AITD) like Hashimoto’s thyroiditis (HT) and Graves’ disease (GD) frequently coexist, entailing numerous potential impacts on diagnostic and therapeutic approaches. Possible correlations might exist through gut microbiota, regulating the immune system and inflammatory responses, promoting autoimmune diseases, as well as shared cytokines in pathogenesis pathways, cross-reacting antibodies or malabsorption of micronutrients that are essential for the thyroid like iron or vitamin D. Vitamin D deficiency is a common finding in patients with AITD, but might protect from autoimmunity by wielding immunoregulatory and tolerogenic impacts. Additionally, vitamin D is assumed to be involved in the onset and progression of CD, presumably plays a substantial protective role for intestinal mucosa and affects the thyroid via its immunomodulatory effects. Iron is an essential micronutrient for the thyroid gland needed for effective iodine utilization by the iron-dependent enzyme thyroid iodine peroxidase (TPO). Despite being crucial for thyroid hormone synthesis, iron deficiency (ID) is a common finding in patients with hypothyroidism like HT and is frequently found in patients with CD. A literature research was conducted to examine the interplay between CD, AITD, vitamin D and iron deficiency. This narrative review highlights the relevant correlation of the two disease entities CD and AITD, their reciprocal impact and possible therapeutic options that should be further explored by future studies.

## 1. Introduction

A substantial number of patients with autoimmune thyroid diseases (AITDs) shows an increased prevalence of coexisting autoimmune diseases [1,2]. Celiac disease (CD) is an inflammatory disease of the small intestine with autoimmune traits [3] that entails intolerance to dietary gluten and might be associated with other organ autoimmunity [4]. The ingestion of gluten triggers chronic inflammation, which leads to villous atrophy, deprivation of brush-border proteins, as well as enzymes needed for the absorption of micronutrients such as iron [5]. In contrast, iron deficiency (ID) worsens preexisting thyroid dysfunction due to the decreased activity of the heme-dependent thyroid peroxidase (TPO) [6]. Diminished levels of iron, folate, vitamin B12, vitamin D, zinc and magnesium are a frequent finding in untreated CD. Deficiencies of various micronutrients frequently coexist and may compromise physical growth and neurological development, as well as raise the risk of morbidity and mortality [7]. Micronutrient deficiencies are associated with a lower quality of life, given various side effects including fatigue, weakness, headache, dizziness or shortness of breath [8,9]. Although a correlation would be biologically plausible, studies yielded conflicting results so far on the relationship of thyroid hormone balance and trace element levels.

Hashimoto’s thyroiditis (HT) and Graves’ disease (GD) are the major causes of hypo- and hyperthyroidism, being mediated by different immunological mechanisms [10]. HT is generally the most prevalent autoimmune disease, frequently clustering with other autoimmune endocrinopathies. The presence of TPO or thyroglobulin antibodies, as well as potentially elevated serum thyroid stimulating hormone (TSH) concentrations can help diagnose the disease. Further, in sonography, a hypoechoic and mostly undersized thyroid gland with inhomogeneous tissue and isolated scarred hyperechoic tissue defines HT. The main feature of GD is circulating TSH receptor stimulating antibodies that bind and stimulate the TSH receptor on thyroid cells, promoting hypertrophy and hyperplasia, eventually resulting in goiter. Patients are predominantly women and may also show high serum concentrations of antibodies against thyroglobulin and TPO [3].

Up to 30% of first-degree relatives of patients with CD and/or AITDs are afflicted by the other disease, respectively. The genes predisposing endocrine autoimmunity, such as diabetes type 1 or AITDs, namely DR2-DQ2 and DR4-DQ8 are substantial genetic parameters of CD, which is an HLA-linked disease as well [11]. CD and endocrine autoimmunity share a similar genetic background. Single nucleotide polymorphisms of several immunoregulatory genes have been found to be overlap susceptibility genes for both CD as well as monoglandular or polyglandular autoimmunity [12]. Genetic overlap between CD and other autoimmune disease may be of clinical relevance, but genetic screening is not yet sensitive nor specific enough to predict the disease onset and progression [13]. Nonetheless, patients with CD should be screened for type 1 diabetes or AITD and vice versa.

Considering the complexity of the mentioned interactions and the partly minor evidence, this review aims to investigate the role of vitamin D and iron, as well as their interplay with the gut microbiota on CD and thyroid function.

## 2. Methods and Material

A literature research was conducted using PubMed and Google Scholar to assess novel, as well as established knowledge concerning the correlation of CD and AITDs, as well as the influence of iron, vitamin D and the gut microbiota on their onset and progression. Main key words were “Celiac disease”, “Hashimoto’s thyroiditis”, “Grave’s disease”, “Autoimmune thyroid disorders”, “iron”, “vitamin D” and “microbiota”, as well as “microbiome”. Eighty-one manuscripts were reviewed; primarily, randomized controlled trials were included but also systematic reviews and meta-analyses that were considered relevant for the subject.

## 3. Vitamin D

Vitamin D is the term of a group of hormones responsible for regulating calcium, magnesium, and phosphate levels in the blood. The majority of the daily requirement of vitamin D is covered by endogenous synthesis, triggered by sunlight/UV-B exposure on the skin, and the remaining part by dietary intake. Calcitriol (or 1,25-dihydroxyvitamin D_3_) is the biologically most active form. It is formed in the kidneys after hydroxylation of calcidiol (or 25-hydroxyvitamin D_3_) by the action of an enzyme called 1-α-hydroxylase [14]. The binding of calcitropic vitamin D to intracellular vitamin D receptors (VDR) in target cells triggers the expression of genes necessary for intestinal absorption of calcium and phosphate, tubular calcium reabsorption in the kidneys, and elevated bone metabolism [15]. Thus, both adequate vitamin D levels in the blood and activity of VDR are crucial for vitamin D signaling and gene expression. In cases of insufficient supply due to lack of sunlight or malabsorption, the use of vitamin D supplements may be required.

### 3.1. Vitamin D and the Immune System

Besides its crucial role in calcium and phosphate homeostasis, a normal vitamin D status protects against respiratory tract infections [16]. Vitamin D is also thought to have preventive effects against other infections and autoimmune diseases, as VDR are also expressed on immune cells like lymphocytes and antigen presenting macrophages [17,18]. In addition, some antigen presenting cells have proven to be able to synthesize vitamin D metabolites in an autocrine/paracrine way. Vitamin D signaling triggers cellular growth and the development of naïve T-cells [15]. It also has a key role in the terminal differentiation of promyelocytes into monocytes, which in turn differentiate into macrophages as part of the myeloid lineage of the immune system [19]. Furthermore, vitamin D appears to have regulatory effects in the production of immunomodulating cytokines to promote inflammatory reactions. This affects immune cell proliferation and differentiation, promoting self tolerance and protective immunity, and thus may prevent the progression of autoimmune diseases. The need for vitamin D in modulating immune responses is undisputed in the literature. Before the discovery of effective antibiotics, patients with tuberculosis were exposed to sunlight/UV-B, triggering the endogenous synthesis of vitamin D and improving outcomes [20].

### 3.2. Vitamin D Deficiency

**Figure 1:**
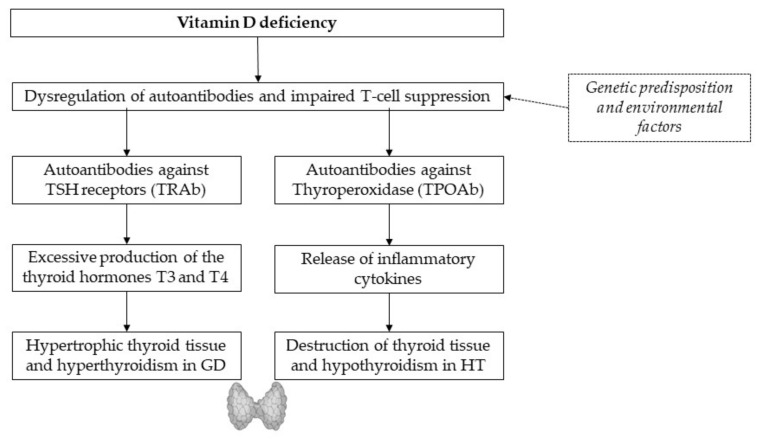
Pathogenic pathway from vitamin D deficiency to AITDs. Further studies are required.

In the current literature, vitamin D deficiency is determined by a calcidiol (or 25-hydroxyvitamin D_3_) level below 50 nmol/L or 20 ng/mL in the blood [21]. Low vitamin D levels are associated with AITDs such as Hashimoto’s thyroiditis (HT) and Graves’ disease (GD) (Figure 1) [18,21]. Both endocrine diseases can be attributed to a genetic predisposition and environmental factors, as well as lymphocytic infiltration and elevated autoantibodies against thyroid tissue [18]. Compared to controls, low vitamin D levels were found more frequently in patients with new-onset autoimmune thyroid diseases [22], as well as inverse relationships of vitamin D and anti-thyroglobulin antibodies (ATAs) [2]. Autoantibodies against TSH receptors permanently stimulate triiodothyronine (T3) and thyroxine (T4) production, leading to enlarged thyroid and hyperthyroidism in GD. Impaired T-cell-suppression due to vitamin D deficiency seems to boost the release of inflammatory cytokines, leading to the destruction of thyroid tissue and hypothyroidism in HT [17]. Oral intake of vitamin D supplements was found to reduce titers of thyroid autoantibodies in levothyroxine-treated women with postpartum thyroiditis. The beneficial effect of vitamin D on autoimmunity may be enhanced by additional selenium supplementation, as a recent study from Krysiak et al. suggested [23].

Patients with AITD display a higher prevalence of coexisting celiac disease (CD). The coexistence of autoimmune diseases may be explained by the common immunopathogenetic mechanisms, as findings of Naiyer et al., suggest the same antibodies affecting both thyroid and intestinal tissue [24]. In CD, the protein gliadin, a component of gluten, triggers proinflammatory gene expression and cytokine release in the intestinal submucosa, leading to chronic inflammation, villous atrophy and impaired absorption of micronutrients such as calcium, vitamin D and iron [25]. Severe iron deficiency worsens preexisting thyroid dysfunction due to the decreased activity of the heme dependent TPO [6], and the lack of calcium and vitamin D has negative effects on bone turnover and bone health.

There is a growing evidence associating vitamin D deficiency with an increased individual susceptibility to other autoimmune diseases [22]. However, the correlations are controversial and require further studies. It is not entirely clear whether low vitamin D levels can be considered a cause or rather a consequence of autoimmune diseases. In GD, effective treatment of the disease did not change vitamin D status, which rather indicates a more causal effect of vitamin D [26].

## 4. Iron Deficiency

Iron is an essential micronutrient that takes part in numerous and important physiological processes in all cells of the human body including oxygen transport and utilization, oxidative phosphorylation, as well as DNA and ATP biosynthesis [27]. Its concentration is regulated via iron absorption in the proximal small intestine by divalent metal ion transporter 1 (DVT1) for non-heme iron and heme iron transporter 1 (HCP1), which also transports folate and is upregulated by hypoxia and iron deficiency (ID) [28,29,30,31]. ID is considered to be the most widespread nutritional deficiency affecting approximately two billion people worldwide [28], being responsible for most anemias. ID anemia (IDA) is the most common extraintestinal finding in patients with CD with a prevalence of about 7% to 81% at the time of diagnosis [5]. In contrast, approximately one in 31 patients with IDA has histologic evidence of CD [32]. Due to permanent inflammation in the small intestines, as well as villous atrophy, patients with CD typically exhibit micronutrient malabsorption and deficiencies [32]. It may even be the only presenting clinical feature of CD in patients without diarrhea or weight loss [33]. Another reason for IDA in CD patients could be systemic inflammation, accordingly high ferritin leading to anemia of chronic disease [34]. Rarely, IDA through blood loss may be present in CD from superimposed small intestinal diseases including neoplasms, ulcerations, gastric or colonic diseases or very scarcely immune-mediated hemolytic anemia with urine blood loss. All these symptoms respond to gluten free diet [33].

Likewise, iron is involved in the maintenance of ideal thyroid function. A considerable amount of animal and human studies indicates that ID—either with or without anemia—compromises thyroid metabolism [35]. Iron is needed for the catalyzation of thyroid hormone synthesis through TPO, which is heme dependent. Additionally, it is required for conversion from T4 to T3, thus if deficient resulting in lower concentrations of circulating T3 and T4 [6]. Many proteins and enzymes involved in the thyroid metabolism are iron and iodine dependent. A systematic review including 57 studies found observational evidence suggesting that iron, selenium and zinc are positively associated with iodine status but data from randomized controlled trials failed to confirm this correlation [6] (urinary iodine does not correlate directly with thyroid function but can be seen as an indicator for the risk of thyroid diseases in a population setting [36]). They also reported no significant effect of iron supplementation on TSH, T3 and T4 [6]. Another systematic review and meta-analysis by Talebi et al., investigating 32 observational studies reported that selenium, as well as zinc, was significantly lower and lead was significantly higher in patients with hypothyroidism compared to healthy controls. Likewise, there was no difference in the concentrations of iron, copper or magnesium between hypothyroid patients and controls [37]. A double-blind controlled clinical trial performed in southern Iran came to the conclusion that treatment with 300 mg ferrous sulfate five times a week significantly increases T4, T3 and triiodothyronine resin uptake, as well as significantly decreases reverse triiodothyronine in comparison to initial values (12%, *p* < 0.001; 3.5%, *p* < 0.001; 16%, *p* < 0.05 and 47%, *p* < 0.001, respectively) [38]. An Indian double-blind randomized intervention study investigated the effect of 190 mg iodine with additional 300 mg ferrous sulphate five times a week with only ferrous sulphate or placebo and concluded that the improvement of iron status is correlated with an improved thyroid function [39]. However, other studies came to conflicting results: Yavuz et al., investigated the effect of iron status on the thyroid hormone profile in school aged children in an iodine-deficient Turkish area and found no link between iron status and thyroid hormone levels [40].

Developing countries in particular are facing the double burden of co-existing high prevalence of iron and iodine deficiency. Zimmermann et al., provided dual fortification of salt with iodine and ferric pyrophosphate and compared the efficiency of the dual- fortified salt (DFS) with that of iodized salt in a 10-month, randomized, double-blind trial in iodine-deficient 6 to 15-year-old children (*n* = 158). After 10 months of treatment, hemoglobin in the DFS group increased by 16 g/L, iron status and body stores increased significantly, and the prevalence of IDA decreased from 30% at baseline to 5% significantly. Moreover, thyroid volume and urinary iodine improved significantly [41]. Subclinical hypothyroidism is associated with diverse side effects such as hypercholesterolemia, infertility or poor obstetric outcomes‚ Specifically, IDA has been reported to be associated with hypothyroidism [42], which is also correlated with low levels of folate or vitamin B12 [43]. The high prevalence of ID in HT patients could as well be a consequence of autoimmune gastritis, a common co-morbidity [2].

Subclinical hypothyroidism should be treated if the patient suffers from IDA concomitantly, according to a randomized controlled trial by Cinemre and colleagues who showed that unresponsiveness of iron replacement therapy could be resolved by thyroid hormone supplementation [42]. A randomized controlled, double-blind trial including 60 patients with hypothyroidism investigated the effect of levothyroxine plus iron salts compared to each treatment alone. The results showed that the increase from baseline levels in hemoglobin and ferritin in the levothyroxine plus iron salt group was significantly higher than in the control group. Additionally, TSH in the study group significantly decreased in the treatment group. These results indicate that treatment with levothyroxine can improve the response to iron salts and that this combination is superior to each component alone [44].

There is a clear link between low levels of certain micronutrients, including iron, vitamin D, selenium, zinc and iodine and thyroid dysfunction. These micronutrient deficiencies might as well contribute to extra-intestinal clinical manifestations of CD, such as neurological symptoms, psychiatric symptoms or bone alterations [45]. These manifold effects implicate the need for a higher awareness of interconnected thyroid and celiac disease and their common micronutrient deficiencies.

Concerning the potential intercorrelations between iron, thyroid disease and CD, the importance of evaluating ID or IDA in CD or AITDs and potential overlaps must be considered. Even though there is conflicting data, ID is a common finding in CD as well as in AITDs. A relevant relationship between ID and these diseases would imply important treatment capabilities.

## 5. Microbiota and Autoimmunity

Novel research suggests that the composition and diversity of the gut microbiota is involved in the onset of autoimmune disorders, such as AITDs, as well as CD [46]. The microbiota is able to affect and regulate the immune system, functions as a “reservoir” for thyroid drugs and is involved in several micronutrient deficiencies, e.g., via absorption. Taking into account these manifold effects on human health, the microbiota could constitute an important link between CD, AITDs and micronutrient deficiencies. Healthy human intestines are colonized by trillions of microorganisms, which co-evolved symbiotically with us, their host, influenced by both, genetics and evolving environment [47,48]. Those microorganisms are mainly bacteria, predominantly *Bacteroidetes* and *Firmicutes* and to a lower extent *Actinobacteria*, *Proteobacteria*, *Fusobacteria* and *Verrucomicrobia phyla*, as a whole representing even an own vital organ for the provision of nutrients and micronutrients like iodine, selenium and iron, the regulation of epithelial development and developing innate immunity [10,47,49,50]. Bacteria and bacterial antigens have been believed to be causally involved in inducing autoimmune diseases for a while. Relevant mechanisms behind their involvement in the onset of autoimmunity include molecular mimicry, where antigen-activated T or B cells are cross-reacting to the body’s own tissue. Epitope spreading, bystander activation in an inflammatory environment during infection and cryptic antigens are further possible models [51]. Gut bacteria generate vitamins such as vitamin K, enable the digestion of insoluble fiber, and refine nutritive, as well as immunomodulatory compounds such as short-chain fatty acids (SCFA) [52]. Microbiota collaborate with physiological processes in the host, including carbohydrate fermentation and digestion, development of gut-associated lymphoid tissue (GALT) and alignment of specific immune responses, as well as the protection from pathobionts [47]. The delivery mode and nutrition through first years of life not only severely influences gut microbiota composition but additionally the onset of autoimmune diseases [53]. There is a higher incidence of infections and an increased susceptibility to allergic diseases after caesarean delivery and formula use, which stresses the strong impact of microbiota on immunity [53,54]. A randomized controlled trial found that aberrant immunoglobulin A (IgA) susceptibility to the gut microbiota precedes asthma and progression of allergies in infants, pointing towards a deteriorated mucosal barrier function in children with allergies [55]. Breastmilk is an essential source of microbes and maternal IgA antibodies for babies and higher microbial richness lowers the risk of developing an allergy during childhood. Aside from that, probiotic treatment of the mother can influence the breastmilk microbiota composition [56].

As mentioned above, gut microbiota is required for the normal function of the immune system, immune system maturation, as well as GALT development, which constitutes 70% of the entire immune system [57] and plays an important role to develop tolerance to autoantigens in the gut mucosa by controlling its toll-like-receptors (TLRs). Microbiota impacts mucus layer thickness, the number of CD4+ T cells and contributes to a healthy epithelial barrier [47]. A breakdown of this epithelial barrier entails an inflammatory cascade, including the secretion of pro-inflammatory cytokines and the engagement of the adaptive immune system [58,59]. This condition is called “leaky gut”, implying an increased permeability of epithelial cells allowing toxins, antigens and bacteria to passage into the blood stream. Growing evidence supports that the microbiota regulate intestinal permeability, which is deteriorated by antibiotics and strengthened by probiotics. Modulation of the microbiota is therefore thought to help alter the course of autoimmunity [60].

### 5.1. Microbiota and CD

The composition as well as the function of the gut microbiota may be associated with the onset and progression of CD. The sophisticated equilibrium between self-tolerance and immunity is regulated by the intestinal epithelial barrier and its intercellular tight junctions [25,61]. In genetically susceptible individuals, this balance may become impaired and intestinal as well as extraintestinal autoimmune disorders can occur. In CD, tight junctions open up, presumably to zonulin upregulation [62] and the antigenic trigger, gliadin, triggers proinflammatory gene expression and cytokine liberation within the intestinal submucosa [25]. Serum zonulin levels reduce immediately, once gluten is removed from the diet. Without gluten, the intestine retrieves its barrier function, auto antibody titers decrease, the autoimmune mechanisms discontinue and, eventually, the intestinal damage heals completely.

The key to the link between gut microbiota and CD may lie in early childhood. The higher prevalence of CD in children born with C-section could be explained through their changed microbiota compared to children born through vaginal delivery, but this remains controversial [52]. Since oligosaccharides of human milk support the growth of beneficial *Bifidobacteria* and prevent the growth of pathogens like *Clostridium difficile* [63], breast-feeding might be an essential part for the engraftment of a healthy, symbiotic gut microbiota. Furthermore, early gastrointestinal infections may favor the onset of CD [64]. On the other hand, an Italian study showed an increase in the prevalence of CD, even after the initiation of rotavirus vaccination [65].

The active state of CD is accompanied by T regulatory cell (Treg) dysfunction, which are normally involved in immune response to antigens and to preserve self-tolerance. It has been shown that cholecalciferol is able to enhance suppressor function of Tregs in patients suffering from diabetes type 1 in a randomized controlled trial by Treiber et al., [66]—similar effects may be expected in other endocrinopathies, or even CD. The nuclear transcription factor forkhead box protein 3 (FoxP3) has been found to be essential for the regulation of Treg-cells and mutations of FoxP3 have been linked to several autoimmune diseases. An imbalance of FoxP3 isoforms, shifted towards a non-functional isoform, investigated in intestinal biopsies seems to be associated with CD. Serena et al., reported the link between alterations in the intestinal microenvironment and host epigenetic alterations were mechanistically connected with immune surveillance. The proinflammatory intestinal microenvironment of CD active patients is enriched in butyrate producing bacteria and may contribute to this disequilibrium of FoxP3 isoforms [67]. HLA-DQ genotype, which contributes to the susceptibility of developing CD, is able to influence the composition of the gut microbiota. A study compared the intestinal colonization of infants with high genetic risk of CD and low genetic risk of CD. High genetic risk was associated with bigger fractions of certain bacterial strains and total gram-negative bacteria count. Higher numbers of affected celiac relatives, especially if the mother is affected, seems to be linked to higher proportions of bacteria like *E. rectale*, *C. coccoides*, *E. coli*, *C. lituseburense* and *Streptococcus-Lactococcus*, that are related to increased risk for CD in children [68]. Children with a genetic risk for CD more prevalently show a higher incidence of pathogenic bacteria such as *enterotoxigenic E. coli* [69]. Sellitto hypothesized that the gut microbiota as a whole, rather than certain infections influence the change from tolerance to immune responses in genetically susceptible individuals. She and her research group investigated longitudinal changes in the microbial strains of genetically susceptible infants for CD from birth to the age of two years. Late exposure to gluten after the age of 12 months was associated with a lower rate of CD autoimmunity compared to early exposure. Metabolomics analysis revealed potential biomarkers for predicting CD. Microbiota of genetically predisposed infants was generally lacking the phylum *Bacteroidetes* but showed an abundance of *Firmicutes*. Moreover, their microbiota did not resemble the composition of adults even at 2 years of age [70].

### 5.2. Microbiota and Thyroid

**Figure 2:**
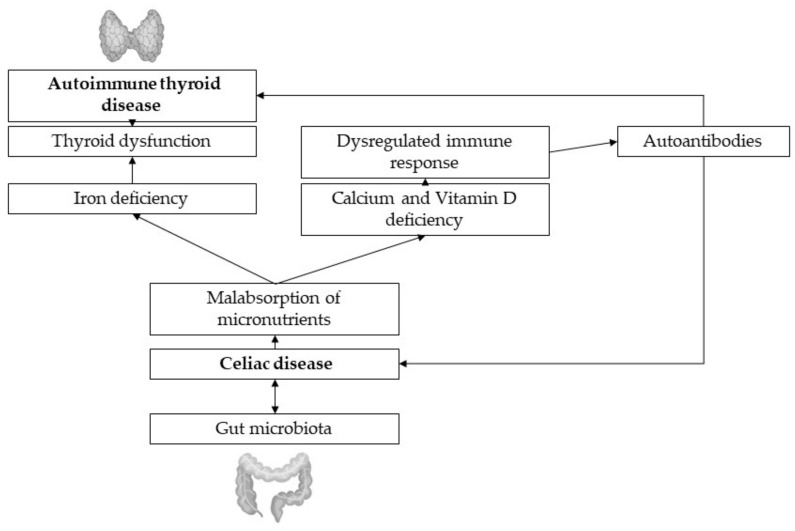
Thyroid-gut-axis.

Research suggests a link between the endocrine system and the gut microbiota. Hormonal levels interrelate with the presence of specific gut microbiota, which is able to produce, secrete, regulate and respond to hormones of the host, affecting not only metabolism and immunity, but also behavior, sexual attraction and appetite [71]. Several studies suggest that in patients with thyroid disorders like HT and GD the composition of the gut microbiota is changed and even increases the diseases prevalence (Figure 2). Microbiota have a remarkable metabolism of thyroid hormones, regulating hormone levels by controlling micronutrient absorption, depletion and enterohepatic cycling [10]. Because of the different underlying immunologic mechanisms in HT and GT, respectively, there may be various roles of the microbiota in these diseases as well. Pathogen-free rats showed an increased susceptibility to HT when microbiota were transferred from conventional rats, supposedly caused by cross-reacting antibodies with TPO and thyroglobulin [72]. Besides, in hypothyroid patients, microbiota diversity is even higher than in healthy controls, which may be due to longer gastrointestinal transit time which is prevalently seen in those patients [10]. High microbial diversity, even if often proposed as beneficial for human health, can entail increased protein catabolism, as well as decreased polyphenol conversion, epithelial turnover and mucus secretion [10,73]. HT patients not only show alterations in their gut microbiota but their microbiota composition is also correlated with clinical parameters, indicating that data about individual microbial composition could help with diagnosis and therapy [74]. In GD, the constitution of the gut microbiota, especially the presence of certain strains like *Paludibacter* and *Allobaculum*, *Limibacter*, *Anaerophaga* and *Ureaplasma* seems to increase susceptibility to disease [75]. Common risk factors for GD like female gender, stress, pregnancy or smoking induce changes in the gut microbiota compared to healthy controls but a definite correlation between dysbiosis and GD has not been found yet [76], even though there are numerous hypothesized mechanisms. Köhling et al., report that remains unclear if bacterial infections can trigger autoimmune thyroid diseases [76].

Numerous organs including the gut are capable of deiodination of T3 or T4. Microbiota seem to have an own deiodinase activity by binding and oxidatively degrading T3 and T4. Through this, they are able to inhibit TSH, and therefore have a direct impact on thyroid hormone levels. Deiodinase activity has been found in intestinal walls of rats [77] and was also identified in the human intestine [78].

L-thyroxine substitution therapy is typically recommended for hypothyroidism. Levothyroxine has a narrow therapeutic index and absorption can be easily disturbed by numerous factors, e.g., if not taken on an empty stomach, or with high fiber or calcium intake. Interestingly, gut microbiota such as microbes like *E. coli* could constitute a reservoir for thyroid hormones by binding it to bacterial thyroid-binding hormone. Taking into account that certain obligate anaerobic bacteria in the gut display glucuronidase activities, gut microbiota research should also focus on thyroxine (T4) metabolism. Through hydrolyzation of conjugated T4 in the intestine, the hormone is able to reenter physiological circulation via the hepatoenteral circulation, joining the iodothyronine pool again [79]. Yao et al., recently showed that the differences in L-thyroxine dose that different patients require to maintain TSH level stability had a relationship with gut microbial composition. Relative abundance of certain strains correlated with thyroxine metabolism were found different between distinct doses of L-thyroxine. Their findings could be due to disparities in the gut´s capacity to metabolize thyroxine. Additionally, they suggested relevant effects of serum cholesterol with L-thyroxine in shaping microbiota. The metabolic resemblance of iodothyronines and bile acid in the intestinal lumen might explain the link between the host´s thyroxine and cholesterol levels [80]. Similarly, Spaggiari et al., showed with his findings that a composite of probiotics containing *Lactobacilli* and *Bifidobacteria* were not directly able to alter thyroid function, but led to less dose adjustments, dose reduction and prevention of serum hormonal fluctuation in the treatment group [81]. Gut microbiota modification through probiotics intake may increase levothyroxine bioavailability and be able to stabilize thyroid function compensation.

## 6. Conclusions

Celiac disease (CD) and autoimmune thyroid diseases (AITDs) like Hashimoto’s thyroiditis (HT) and Graves’ disease (GD) frequently coexist, entailing numerous potential impacts on diagnostic and therapeutic approaches. Accumulating data supports the existence of a significant thyroid-gut-axis, indicating effects of the gut microbiome not only on the immune system and the absorption of micronutrients, but also on thyroid function. Micronutrients such as iron and vitamin D often lack in CD, but also frequently in thyroid diseases, implicating intercorrelating mechanisms. This interconnected synergy can be easily disturbed by numerous events, including environmental factors, early infections, birth mode or eating habits.

There is a higher prevalence of coexisting thyroid and gut related disease, including HT and GD, as well as CD—and dysbiosis frequently co-occurs in this context, either. An altered microbiota is able to change the immune response as well as onset of autoimmune diseases and it is probably able to function as a reservoir for thyroid hormone medication. Supported by a proper composition of the gut microbiota which binds it to bacterial thyroid-binding hormone, patients could reduce hormone fluctuations and reduce their dosage of L-thyroxine. The role of microorganisms and microbiota in the development and progression of AITDs and CD is still controversial and needs to be further elucidated. However, increasing evidence suggests the importance of this thyroid–gut axis, which is thought to modulate autoimmune disorders. Patients often refer to changes in their quality of life and thyroid function in relation to dietary changes. Probiotics could represent a novel additional treatment option for patients with need for thyroid hormone substitution.

There is a clear link between the lack of micronutrients such as iron and vitamin D and CD, as well as AITDS. Iron deficiency is a common finding in CD, presumably as a result of permanent inflammation and villous atrophy. ID occurs often in thyroid diseases as well and deteriorates preexisting thyroid dysfunction, for example, through inhibiting the activity of heme dependent TPO. Studies still yield conflicting results, but a correlation between appropriate iron status and proper thyroid function appears to be clear.

Vitamin D is often lacking in patients with AITDs but might protect from autoimmunity by wielding immunoregulatory and tolerogenic impacts. Vitamin D deficiency is associated with AITDs such as HT and GD, but the correlations are controversial and require further studies. It is not entirely clear whether vitamin D deficiency can be considered a cause or rather a consequence of autoimmune diseases, even though studies point towards a rather causative role of vitamin D.

Future studies should try to examine if and how a gluten-free diet can prevent or delay the development of CD and endocrine autoimmunity of children at risk. The manifold consequences implicate the need for a higher awareness of interconnected thyroid and celiac disease and their common micronutrient deficiencies. There is a close relationship between CD and endocrine autoimmunity, which justifies broader immune genetic and endocrinological screenings of celiac patients.
